# Biphasic patterns of diversification and the emergence of modules

**DOI:** 10.3389/fgene.2012.00147

**Published:** 2012-08-07

**Authors:** Jay Mittenthal, Derek Caetano-Anollés, Gustavo Caetano-Anollés

**Affiliations:** ^1^Department of Cell and Developmental Biology, University of IllinoisUrbana-Champaign, IL, USA; ^2^Evolutionary Bioinformatics Laboratory, Department of Crop Sciences, University of IllinoisUrbana, IL, USA; ^3^Institute for Genomic Biology, University of IllinoisUrbana-Champaign, IL, USA

**Keywords:** diversification, biphasic hourglass, linkage, competitive optimization, module

## Abstract

The intricate molecular and cellular structure of organisms converts energy to work, which builds and maintains structure. Evolving structure implements modules, in which parts are tightly linked. Each module performs characteristic functions. In this work we propose that a module can emerge through two phases of diversification of parts. Early in the first phase of this biphasic pattern, the parts have weak linkage—they interact weakly and associate variously. The parts diversify and compete. Under selection for performance, interactions among the parts increasingly constrain their structure and associations. As many variants are eliminated, parts self-organize into modules with tight linkage. Linkage may increase in response to exogenous stresses as well as endogenous processes. In the second phase of diversification, variants of the module and its functions evolve and become new parts for a new cycle of generation of higher-level modules. This linkage hypothesis can interpret biphasic patterns in the diversification of protein domain structure, RNA and protein shapes, and networks in metabolism, codes, and embryos, and can explain hierarchical levels of structural organization that are widespread in biology.

## Introduction

In evolution, a pattern of change may recur in diverse contexts. Classic examples include punctuated equilibrium, with alternation of stasis and rapid change; prolonged trends of increase in size; adaptive radiation; convergence; and mass extinction. It is an interesting challenge to understand how a pattern of change arises. Can it only arise in one way, or are alternative paths possible? If the latter case is true, what are these paths, and in what circumstances are they likely to occur?

**Figure 1:**
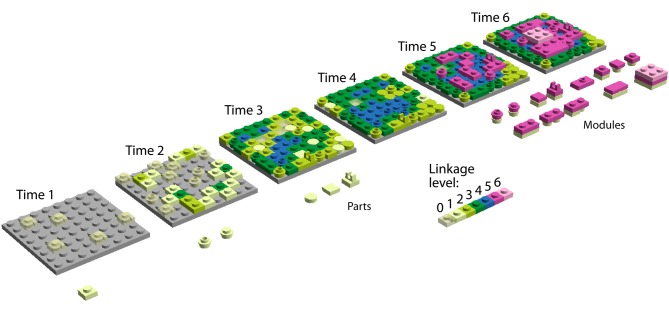
**A linkage model of emergence of modules.** We illustrate our main hypothesis—diversification and integration of parts unify parts into modules, which then diversify—with the evolution of a system of Lego® blocks. The blocks represent aspects of hierarchical levels of organization in the system. Stud-hole interactions of neighboring blocks and interactions of blocks with walls should in reality be portrayed as multidimensional. Biologically, levels 1, 2, 3, and 4 could represent (for example) levels of organization within proteins—spaces of protein sequences, structures, domains, and quaternary structures, respectively. These levels can be seen as multidimensional spaces in which parts diffuse as they change. We show only three levels as these evolve, with each level materializing in two dimensions—a layer—for the sake of simplicity. Blocks interact only with neighbors and attempt to maximally occupy a space defined by their functionality. Hierarchical level 1 (grey base plate) is a previous space that we will not describe. Hierarchical level 2 develops an increasing repertoire of parts (blocks of different shape in shades of green and blue) and an increasing number of interactions per part (linkage). This increases the connectivity and diversity of parts, with sides of the blocks in contact with others representing extents of linkage 0, 1, 2, 3, and 4. Linkage increases with interactions among blocks; color hues of blocks represent extent of linkage. Hierarchical levels 3 and 4 are only accessible to blocks when linkage extent 4 (dark pink) is achieved, constraining change at levels 2 and below (structural canalization). These interactions enable new extents of connectivity among blocks within and between hierarchical levels and result in modules. In the figure, new block parts and modules in the system are enumerated below the base plates as they appear for the first time in the time series. This enumeration of novelties shows a clear hourglass pattern, which stems from: (1) increasing linkage and limits of space accessibility for blocks due to competitive optimization—this results in percolation in the network of interacting parts, and (2) the rise of modules and new levels of diversification, which increases the evolvability of the system. Note how the discovery of parts reaches a peak, then decreases to zero (hourglass constriction and emergence at time 4) and finally explodes in a combination of modules once hierarchical level 3 is established. Note also how easy it is then to establish hierarchical level 4 (light pink). The basic premise is that a new hierarchical level can only be added if linkage has increased to levels that originate modules (networks of block parts enriched in blue or dark pink). In our model, mutation sometimes generates parts that block formation of modules and top hierarchies (blocks with protrusions or without studs or holes). Competitive optimization and linkage also occur in hierarchical levels 3 and 4 but are not showcased. These additional hourglasses are all interlinked to each other through processes of “sandwiched emergence.”

Diversification occurs throughout evolution, encouraging us to look at its patterns of change. We focus on biphasic patterns of diversification, in which diversity decreases to a minimum and then increases again. The stimulus for our inquiry was the work of Sander (1983), Duboule (1994), and Raff (1996) on developmental hourglasses—biphasic patterns of diversification in the development of embryos. These studies interpreted such patterns in terms of linkage, the extent of interaction among parts of a system. In this paper we propose a general linkage hypothesis to explain evolutionary biphasic patterns that exist at many levels of biological organization. Note that our hypothesis is novel, and different from the proposed developmental hourglasses, in ways that will be explained below. In our hypothesis, a system with many parts can have alternative associations and functional capacities. Through mutation and reassortment the parts become more numerous and diverse. With selection for a specific association or capacity, the system undergoes *competitive optimization*: The parts interact more strongly, competing, and cooperating to meet the selection criterion. That is, linkage among the parts increases, as does the organization of the system. As functional niches within the organization become filled, fewer new parts survive competition, and the rate of diversification of parts decreases. Increasing linkage shapes modules—sets of parts that interact more strongly with each other than with other parts of a system. Since linkage is tighter within a module than between the module and its context (Simon, 1962), modules become free to diversity in different contexts within the system and in various ways (e.g., by producing new kinds of variants or by linking to other modules to form higher-level modules). This development of autonomy produces a second phase of diversification of parts. Figure 1 illustrates the principle with a simplified model.

In the next section, we present examples of this hierarchy-generating process in the evolution of macromolecules and networks. We first describe patterns of structural diversification of proteins and nucleic acids. We then focus on biological networks, dissecting patterns in (1) emerging metabolic networks during origins of life, (2) emerging biological codes during the rise of diversified lineages, and (3) at the interface of evolution and development.

## Biphasic patterns in the diversification of macromolecules

The sequence and structure of proteins, nucleic acids, and other polymers used by biological systems to function and to store information diversify in various ways. For example, biphasic patterns of diversification are evident in the evolution of protein structures and of other macromolecules.

### Diversification of protein structures

Proteins are made up of one or more *protein domains*, compact folding units of molecular structure and function. Protein domains recur in life and represent evolutionary units. They are structurally and functionally diverse, and they interact with small and large molecules (including other domains, metabolites, lipid bilayers, and nucleic acids) to function in diverse cellular processes. The Structural Classification of Proteins (SCOP) organizes related protein domains into hierarchical levels of structural organization (Murzin et al., 1995; Andreeva et al., 2008). The *fold family* (FF) level describes domains that are closely related at the sequence level (>30% pairwise amino acid sequence identities) or that share similar structures and functions despite lower sequence identities. The *fold superfamily* (FSF) level pools domains with similar structural and functional features that suggest probable common ancestries. The FSFs of this level can group one or more FFs without a formal structural definition. The *fold* (F) level defines domains that have common 3-dimensional molecular topologies (architectural designs). Their similarity may manifest the physics and chemistry of folding rather than an ancestral relationship.

**Figure 2:**
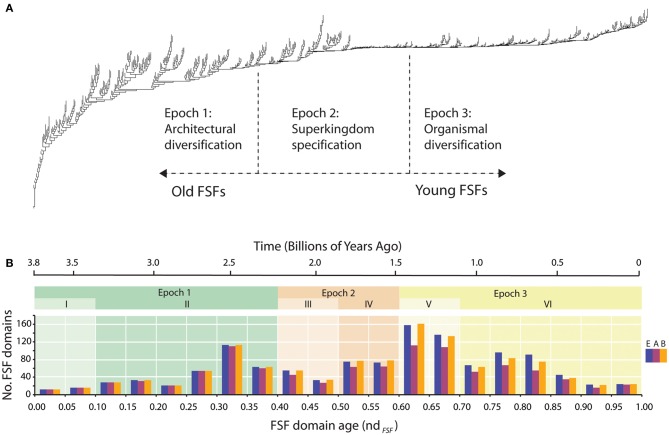
**Phylogenetic trees of protein domain structures.** Phylogenetic trees of protein domain structures. **(A)** A tree describing the evolution of groups of domains was reconstructed from a genomic census of domains in hundreds of genomes. The approach was reviewed in Caetano-Anollés et al. (2009a). The leaves of the tree (taxa) are FSFs. The distribution of FSFs among superkingdoms Eukarya (E), Archaea (A), and Bacteria (B) is remarkably consistent (Wang et al., 2007; Wang and Caetano-Anollés, 2009). The most ancient FSFs are present in all organisms, but as time unfolds, FSFs are first lost in emerging archaeal lineages, and then in eukaryal and bacterial lineages. Superkingdom-specific FSFs only appear quite late in evolution. This “taxonomic” distribution of FSFs in life define the three epochs of the protein world that are mapped to the tree: Epoch 1, a period in which ancient molecules emerged and diversified while keeping proteomes similar to each other in a largely communal world; Epoch 2, a period in which molecules sorted in emerging organismal lineages and some became specific to emerging superkingdoms; and Epoch 3, a time in proteome diversification in a clearly “tripartite” world. **(B)** Number of FSFs appearing during a time interval (bin) vs. age of the interval. Bars in each bin represent the number of novel FSFs in each superkingdom. Time is given as node distance, nd_FSF_, a statistic that is derived directly from the tree of structures (which is rooted). Because the trees are highly unbalanced and the timing of discovery of domains is largely defined by molecular speciation (i.e., by the shape of the trees) and not by changes of domain abundance (i.e., by the length of branches) (Wang et al., 2011), the relative number of internal nodes in lineages (nd_FSF_) from the root to each leaf of the tree can be considered a good proxy for time. nd_FSF_ thus defines an age of domains and a molecular clock, with nd_FSF_ = 0 representing the origins of protein FSF domains(the P-loop hydrolase FSF) and nd_FSF_ = 1 the most recent FSFs that appeared in protein evolution. The three Epochs of the timeline are shaded and are divided into six phases (I–VI) according to Wang et al. (2007). The molecular clock of FSFs (Wang et al., 2011) places the relative timeline in a geological time scale.

The age of a group of protein domains defined at a particular hierarchical level of structure (e.g., the age of a fold) is the time interval from the origin of the founder of the structural group to the present. For example, the age of the P-loop hydrolase fold, the most ancient protein group, is ultimately defined by the oldest domain belonging to that fold defined at F, FSF, or any other level of structural abstraction. Such ages can be estimated from phylogenetic trees that describe the evolution of domain structures (Caetano-Anollés and Caetano-Anollés, 2003). In a tree with organisms as taxa (trees of species), the distribution of members of the group among organisms suggests the branch of the tree in which the founder evolved. This approach to estimating ages has been recently used in genomic phylostratigraphy of metazoan species (Domazet-Lošo et al., 2007). However, many groups of domains have founders that are universal and are phylogenetically uninformative, since they can only be traced to the basal branch of the universal tree of species (sometimes referred to as the “tree of life”). A tree with groups of protein domains as taxa provides a direct estimate of domain age for all domains (recent or ancient). These trees are analogous to trees of genes, but instead of defining the evolution of entire gene products, the trees describe the evolution of parts (molecular domains). The tree can be reconstructed from a census of the occurrence and abundance of domains in proteomes. Such trees have been derived from a protein census at FF (Caetano-Anollés et al., 2011), FSF (Wang et al., 2007), and F (Caetano-Anollés and Caetano-Anollés, 2003) levels of structural abstraction. Figure 2 shows an example of such a tree, with branch lengths indicating change in domain abundance and branch leaves representing all domains that are known. The tree is rooted and its topology determines the evolutionary age of each domain. Correlation of node position in the tree with other data for dating structures shows that a molecular clock exists (Wang et al., 2011). Thus, the age of each domain can be placed in a true chronological timeline that spans ~3.8 Gyr (billions of years), assuming all domains follow the clock-like pattern. While this may not be true for all domains (the clock may tick differently for different domain groups), the general pattern holds for the entire set of domains (Wang et al., 2011). Distributions along the timeline show a clear biphasic pattern of diversification in the rate of appearance of FSFs (Figure 2B), the rate of appearance and sharing of FSFs in Gene Ontology categories (Caetano-Anollés et al., 2011), the number of functions in single and multidomain proteins that are encoded in human and plant genomes (Wang and Caetano-Anollés, 2009), the number of FSFs per fold (Caetano-Anollés et al., 2011), the number of FFs per FSF (Kim and Caetano-Anollés, ms. in preparation), and the abundance of genes per corresponding domains (Nasir and Caetano-Anollés, ms. in preparation).

How can we explain these patterns? Consider structural variants of FSF domains that are produced by mutation of protein-coding genes, often after duplication and divergence of a coding region. The most primitive FSFs must have been formed with high propensity (were highly favored in an energetic landscape), performing few functions with low speed and catalytic specificity. The cytosolic content of cells is by definition far from an ideal solution, tightly packing proteins, nucleic acids, and other macromolecules (Ellis, 2001). There are strong reasons to believe that this “macromolecular crowding” existed already in primordial cells and constrained the functional niches that existed in the cell. These niches however diversified with the discovery of new ecological niches as geochemistries unfolded in the changing landscape of Earth. New FSFs could survive if their proteins populated new functional niches of the cells, or took over previously occupied niches by catalyzing reactions faster or more specifically than other enzymes (Ycas, 1974; Kacser and Beeby, 1984). Within this context, proteins initially occupied the space of functional niches sparsely. Consequently, there was little interaction among FSFs beyond the formation of functional networks; their linkage was weak. However, as proteins diversified in structure and function, competition among FSFs to perform a given function increased. Furthermore, there was increasing selection for cooperation within a cell, as enzymatic pathways, assemblies of macromolecules, and gene regulatory networks evolved. FSFs would have differed in their capacity to work well together in this organization. And, cells with different repertoires of FSFs competed for ecological niches as the cells interacted with the environment. This competition favored some assemblages of FSFs at the expense of others. Thus, competition among FSFs for functional niches within cells, selection pressure for cooperation within cells, and competition among cells for ecological niches all tended to increase the linkage among proteins and the structural organization of cells. As a consequence, increasing linkage decreased the rate of survival of new FSFs.

During competitive optimization parts link to form modules, which then may diversify in various ways (Caetano-Anollés et al., 2009a). Lower-level modules can combine diversely to form higher-level modules, in a hierarchy. Proteins evolved through the assembly and integration of submodules at several levels, including amino acids, secondary and suprasecondary structures, domains, domain combinations, homomers in quaternary structure, units of macromolecular complexes, and subnetworks in metabolism and signaling (Pereira-Leal et al., 2006). The hierarchical nature of submodule and module integration is made explicit by combining submodules such as amino acids into diverse secondary and suprasecondary structures and these into wide range of domains and domain combinations through covalent bonding. Homomers can be similarly combined into quaternary structures and complexes through non-covalent bonding or through interaction via intermediate molecules. Some aspects of these hierarchies are made explicit in bioinfomatic constructs, including efforts of classification of structure and function in proteins. Linkage can increase in parallel at all of these levels of organization as cells evolve, following patterns of “sandwiched emergence” that have been described for the emergence of complex societies (Lane, 2006).

Linkage among parts increases during physical phase transitions such as crystallization and magnetization. Eigen (2000) suggested that natural selection is a phase transition in an information space. The formation of a module through competitive optimization may be a phase transition in a system far from equilibrium (Hinrichsen, 2006). Cooperative interactions among the parts make the transition autocatalytic or self-promoting. For example, diversifying FSFs created new functional niches, in which more FSFs could occupy and survive (Schmidt et al., 2003). Thus, as competitive optimization proceeded, the increasing density of the population of occupied niches further increased, until potential niches became saturated. Such saturation resembles the occupation of all binding sites in a layer of a growing crystal. In other words, increases in “niche occupancy” (an ecological concept) are connected to processes of saturation and crystallization (a physical concept). Note that borrowing from ecology and physics is appropriate. In ecology the concepts of the niche (how an organism makes a living) and competitive exclusion (one species-one niche) delimit the interplay between abundance of a species and its range within a region but also underlie the evolutionary emergence of self-organized clumps of species (Gravel et al., 2006; Scheffer and van Nes, 2006). In physics, crystallization explains the formation of crystals once solute molecules start to cluster into nanometer scale nuclei that beyond a threshold are stable and do not redissolve. These paradigms help explain a critical point in the saturation process that is induced by the process of competitive optimization.

The second phase of FSF diversification proceeded with divergence of the three superkingdoms of life (Wang et al., 2007; Wang and Caetano-Anollés, 2009). A “big bang” of architectural innovation in Eukarya and Bacteria may have resulted from novel functional niches and novel processes for generating new FSFs. Wang and Caetano-Anollés (2009) proposed that during the second phase, an explosion of combinations of domains in proteins resulted from novel genomic rearrangement mechanisms, perhaps mediated by chromosomal recombination, intronic recombination of domain-encoding exons and faulty excision of introns, domain insertion and deletion at C and N termini, retrotransposition, and “exonization” of intron sequences. While the appearance of novel proteins enabled these processes, it is evident that the protein landscape increased significantly its diversification potential (Wang and Caetano-Anollés, 2009).

As modules emerged in molecules, cellular organization became more and more modularized, with cellular machinery being constructed from the molecular modules. Modularization of cellular architecture facilitated multicellular organization. The advent of multicellularity provided novel functional niches for FSFs. After the minimum rate of FSF generation was reached, cells formed a plethora of multicellular organisms through modifications of embryogenesis, with accompanying elaboration of diverse proteins involved in cell–cell communication (recognition, affinity, signaling, and defense; Caetano-Anollés and Caetano-Anollés, 2005). Multicellular eukaryotes offered many new niches for diversification of organisms and their FSFs. Archaea probably received some of the new FSFs through lateral gene transfer. This scenario is compatible with the predominance of second phase diversification in Eukarya and Bacteria, evident in Figure 2B. From the second peak of diversification to the present, the rate of FSF appearance declined. Competition among FSFs may have inhibited the successful introduction of new FSFs and favored instead their extensive reuse as modules.

Thus the linkage hypothesis can explain a biphasic pattern of FSF diversification. Competitive optimization among a diversifying set of interacting proteins produced a module, the network of protein-mediated processes in ancestral cells. In these cells new possibilities for diversification arose and were used. As we will now show, the linkage hypothesis can explain evolutionary patterns in individual macromolecules.

### Competitive optimization of the shapes of macromolecules

A macromolecule evolves through a biphasic distribution of molecular shapes. For example, Ancel and Fontana (2000) modeled the formation of secondary structure in RNA, treated as convenient planar abstractions of three-dimensional folds. Within the range of free energies accessible at a given temperature, an RNA molecule may fold into diverse shapes. This “plastic repertoire” represents an ensemble of possible conformations. If shape determines molecular function and function impacts on the fitness of an organism, the more time an RNA spends in favored shapes the greater its impact on the organism's fitness. If selection favors a target shape within the plastic repertoire, mutants of the RNA sequence can optimize folding to that shape. The mutant RNA sequences that tend to survive this selection have fewer thermally accessible shapes, and most of these resemble the target shape. These shapes are more stable, so RNAs will spend more time in them. During selection the variability of shapes under point mutation also decreases; most of the mutants fold to nearly the target shape. That is, lock-in or canalization to the target shape occurs. This process is autocatalytic in that increased occurrence of the target shape confers a selective advantage, which increases the fraction of the population having the associated RNA sequences, and so makes further improvement likely.

**Figure 3:**
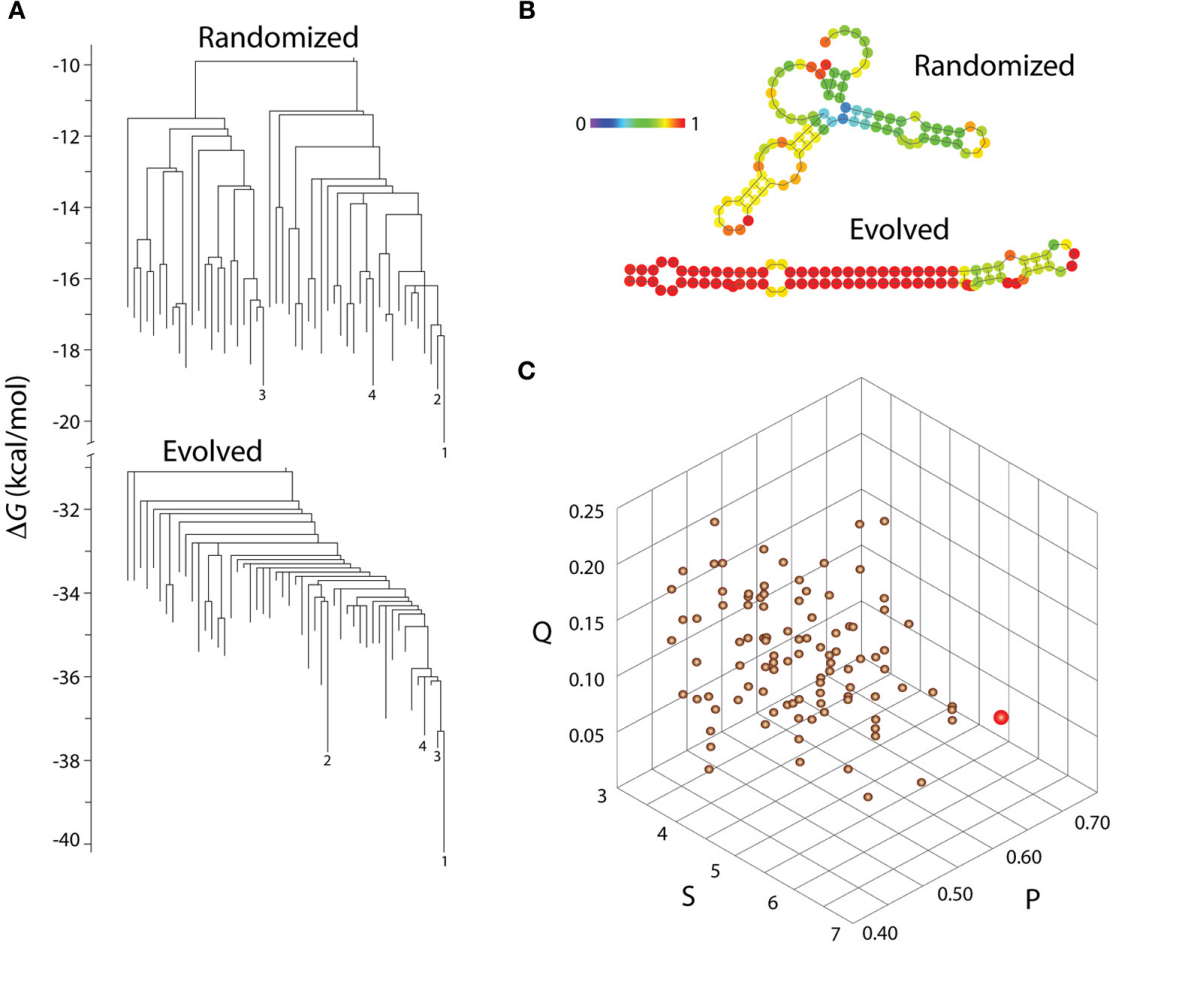
**Evolution of molecular shapes. (A)** Barrier trees describing the low energy portion of the free-energy landscape of a naturally evolved molecule, let-7-a-1, a micro RNA stem loop from zebrafish (*Danio rerio*) that is 89 nt long (Chen et al., 2005), and its randomized derivative. Barrier trees describe the likelihood of conversion (at constant temperature) of one shape configuration into another, with leaves representing shapes (macrostates corresponding to local energy minima), branch lengths (vertical dimension of the tree) representing free energy (Δ G), and internal nodes connecting branches representing energy barriers (basins) that limit transformation between shapes. The randomized structure is one out of 100 obtained using OMROKGEN (Knudsen and Caetano-Anollés, 2008) and shows a more degenerate energetic landscape. Barrier trees were obtained with the software Barriers (Flamm et al., 2002) and computed with the Vienna RNA webservers (http://rna.tbi.univie.ac.at/). **(B)** Minimum free energy secondary structures of the evolved microRNA and its randomized derivative. Nucleotides (depicted by circles) are colored according to the probabilities of being paired in paired regions and unpaired in unpaired regions (probabilities increase from blue to red). **(C)** A morphospace delimited by statistical parameters of the molecules shows that the evolved sequence (red circle) is more ordered and less frustrated than the cohort of 100 structures with permuted sequences (copper circles), which show only the effects of self-organization. Parameters Q (Shannon entropy of the probability matrix), P (base pair propensity), and S (mean length of helical stems) are described in Schultes et al. (1999) and were calculated using STOAT (Knudsen and Caetano-Anollés, 2008). Q measures conflicting inter- and intra-molecular interactions during RNA folding and P and S describe how extensively folded and ramified are the folded molecules. Randomization of sequences decreases the thermodynamic likelihood of base pairing during the energy minimization process of folding (it increases Q) confirming the “structural canalization” lock-in effect of target shapes during molecular evolution.

For macromolecules, a free energy landscape characterizes the kinetics of folding along a morphogenetic trajectory. In this landscape a canalized sequence has low barriers among many shapes with a relatively high minimum free energy (Figure 3). Folding proceeds down a funnel to a single shape with low minimum free energy, the target or native shape. The minimum free energy of a macromolecule's shape corresponds to the linkage within it, the extent of bonding among its monomers. Thus, from an initial diversity of plastic shapes, sequences and morphogenetic trajectories, selection funnels RNA sequences in a genetic neighborhood to the favored target shape, which has a low free energy and high linkage. This shape is a robust module. Although, the target shape is insensitive to point mutation, it is evolvable; subsequent diversification of sequences and shapes may occur through recombination or under new selection pressures. Wagner (2008) showed that robustness and evolvability, suitably defined, can be synergistic. Aiding this second phase of diversification, the canalized shape is modular, in the sense that it contains context-insensitive submodules that can evolve relatively independently of each other.

**Figure 4:**
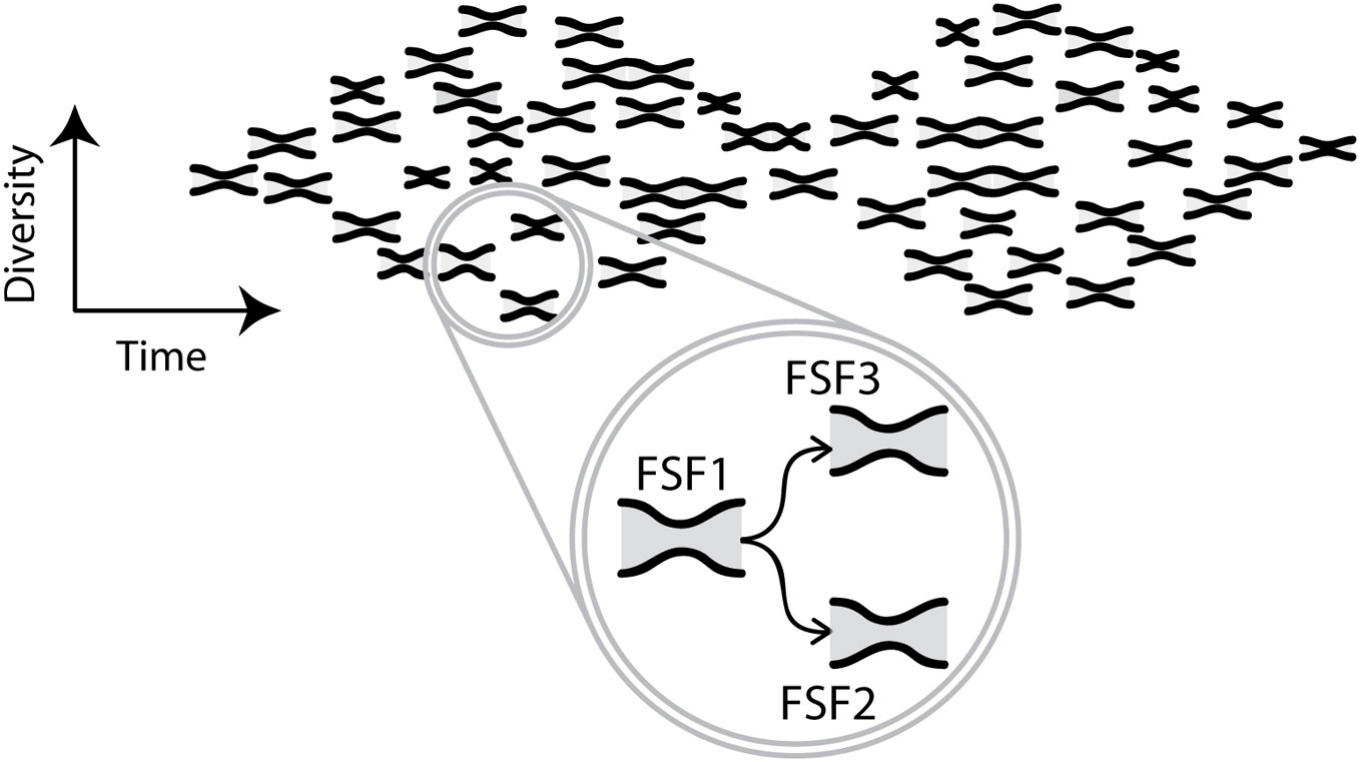
**Evolution of biphasic patterns.** The cartoon illustrates a network of biphasic patterns of diversification of FSFs as the network of structures evolves in time. The horizontal axis represents time and the vertical axis represents diversity. Each small hourglass describes the evolution of a different FSF, through convergence from more to fewer variants of the FSF, with subsequent diversification of its variants. For each small hourglass, the latter phase of diversification continues to the present, though this continuation is not shown in the cartoon. The extent of convergence and divergence, and the temporal scale, may vary among small hourglasses (not shown). Since, this interplay of diversity and time defines a multidimensional landscape it is difficult to visualize. Moreover, fewer small hourglasses are shown than the thousands of actual FSFs. In the circle, three labeled small hourglasses are enlarged to show that FSF2 and FSF3 originate from FSF1. All small hourglasses have evolved from the left-most one, but the only origins shown are those indicated by arrows in the circle. Arrows represent wander sets of protein sequences diffusing by mutation in a multidimensional space of protein sequences with random walks resulting in the origination of a new FSF. During the first phase of the new small hourglasse, the initial mutant form is elaborated into variants—a widening diversity not shown. These variants compete for the same functional niche, so their diversity decreases toward the narrow neck of the hourglass. After this neck, variants can diversify because they can occupy other niches. Collectively, the small hourglasses form a biphasic pattern which represents the biphasic pattern of FSF appearance shown in Figure 1B.

It is likely that this scenario also describes the evolution of proteins. Models of protein folding show that typically the native shape is relatively insensitive to mutations, and a free energy funnel directs folding to this shape, which is robust to environmental change (Taverna and Goldstein, 2002; Wroe et al., 2005). Presumably each FSF evolves through biphasic diversification: mutations can enable an FSF to preferentially adopt a new shape within its plastic repertoire. Mutation with selection for this shape could reduce plasticity and deform the free energy landscape, producing a new funnel that folds mutant sequences to the new target shape. Further mutation could diversify the proteins having the new FSF. Thus, the biphasic pattern of diversification for FSFs collectively, presented above, is a network connecting biphasic patterns for the individual FSFs (Figure 4). In this network the second divergence phase for an earlier FSF becomes the source for the first phase of a later FSF. The pattern in Figure 4 applies to domain groups at all levels of structure.

## Competitive optimization in the evolution of networks

Networks of macromolecules underlie the operation of cells and organisms. We now discuss how competitive optimization may have helped to generate two intracellular networks, metabolism and coding in translation, and multicellular networks in the development of embryos and in epigenetics.

### Competitive optimization in the very early evolution of metabolism

Alternative networks that perform the same function, some better than others, may evolve and compete to optimize functioning. For example, Wächtershäuser (1990) and Morowitz (1999) proposed that the reductive citric acid cycle self-organized abiotically. Diverse alternatives to the citric acid cycle are possible, but the naturally occurring network has the most favorable combination of traits—it uses fewer steps and produces ATP at a greater rate than most alternatives, and it is especially favorable in other respects (Meléndez-Hevia et al., 1996). Thus, competition among such alternatives, operating in the reductive direction, may have occurred during self-organization of the cycle.

The cycle is autocatalytic in that it produces more of its own intermediates; running the cycle with carbon dioxide and one succinate molecule produces two succinates. Thus, alternative uses of the cycle's intermediates are possible, allowing a new phase of diversification (Mittenthal et al., 2001). Such uses would have progressively enlarged the metabolic network, as minerals and organic molecules, including products of the network, catalyzed the formation of sugars, fatty acids, lipids, amino acids, and nucleic acids. Subsequent rounds of competitive optimization may have occurred: Morowitz (1999) proposed that the metabolic network evolved as a sequence of shells, with a gateway reaction giving access to each new shell. In this view, a transaminase was the gateway for synthesis of amino acids from metabolites that were produced in the core, which contained the reductive citric acid cycle. Phylogenomic analysis of the structure of metabolic enzymes supports this shell scenario (Caetano-Anollés et al., 2009b). Molecular canalization may have locked in the transaminase function. Thus, very ancient metabolic networks may have evolved through a network of processes that at a later time enabled the biphasic patterns generating FSFs and individual proteins.

### Competitive optimization in the evolution of codes

Codes are biases that exist in systems. There are many biological codes (Barbieri, 2008). A model for the evolution of the triplet code for translation (Vetsigian et al., 2006) shows the role of competitive optimization. This model rests on a proposal by Woese (1998, 2002) for the evolution of the universal ancestor of life. In this view, communities of early cells competed for limited cellular resources. Within a community, high mutation rates and rampant lateral transfer dominated the transmission of information, overwhelming vertical transmission and aborting the rise of diversified organismal lineages. Cells readily exchanged parts. Communities in which new parts improved function preferentially survived. Among these improvements, protocols that facilitated the sharing of innovations, such as, the genetic code, would allow more sharing, better performance, and more rapid communal growth. Within a community, an optimized genetic code would enable more efficient protein synthesis and more stable proteins, facilitating lateral transfer of proteins and translation mechanisms. These transfers could accelerate the use of the code and the growth of the community, speeding its rise toward dominance. Competition among communities using different genetic codes should favor growth of larger communities with more optimal codes, in which more innovations would probably be generated and more extensive sharing was possible.

Thus, a positive feedback loop evolved in which lateral gene transfer promoted more similar and better-functioning codes and translation mechanisms, and *vice versa*. This loop promoted autocatalytic growth of communities. Sharing between cells of a community tended to standardize interactions within and among the cells' subsystems. The accuracy of translation and replication increased. The complexity and specificity of linkages within cells increased, in a process resembling crystallization. Tight linkage made subsystems resistant to further modification through lateral transfer of molecular information. Rates of mutation decreased. Thus, vertical inheritance could become the predominant mode of transmission. A Darwinian transition occurred, from collective evolution within communities of cells to species of cells evolving largely in parallel. A biphasic pattern is evident here—cells share diversifying parts, but competition among communities leads to standardization and increased linkage of parts. Consequently a new phase of diversification becomes possible: distinct lineages with limited interaction through lateral transfer arose and now embody a universal tree of cellular life.

This pattern of cellular evolution is coupled to the biphasic pattern of FSF evolution. As proteins evolved, the growing set of FSFs would have included proteins that could improve protein synthesis, speeding the more efficient generation of proteins, which folded more efficiently but were also more diverse. It is noteworthy that the translation machinery underwent a kind of crystallization at the peak of the first phase of FSF diversification (Caetano-Anollés et al., 2011, 2012). At this time a fully functional peptidyl transferase center emerged (Harish and Caetano-Anollés, 2012) and organismal diversification began (Kim and Caetano-Anollés, 2011).

### Competitive optimization in the evolution of development and epigenetics

In the development of an embryo, linkage is manifest in the connectivity of signaling networks, gene regulatory networks, and networks of interacting proteins. These change the state of differentiation and aggregation of macromolecules and cells to build the structure of the embryo. Kirschner and Gerhart (2005) proposed that development evolved through a process called facilitated variation. Linkage increased within each of a set of core processes, generating reusable modules. These could be coupled together in diverse ways, allowing flexible and robust variation in development.

Such reorganization is evident in the response of organisms to a new selection pressure. Throughout embryogenesis, canalization stabilizes the normal development of tissues and organs within a range of genetic or environmental variations. A new selection pressure may elicit diverse changes in development within the physiological repertoire of the embryo. Some of these changes may be adaptive, increasing fitness under the new selection. If this pressure is sustained, organisms with genomes altered by mutation and reassortment of genes will compete to generate an adaptive modification as standard equipment. After selection the novelty may still develop without the perturbation (Waddington, 1942; McLaren, 1999). This phenomenon, genetic assimilation, can be interpreted as manifesting a course of development that is normally silent but is made accessible through mutations that channel development to a new target. Rutherford and Lindquist (1998) showed that a heat shock protein, Hsp90, contributes to the silencing. In *Drosophila*, many abnormal structures develop when mutation or chemicals reduce chaperoning by Hsp90. Abnormalities that are targeted for selection continue to develop after Hsp90 is again normal. After selection, presumably the abnormal morphogenetic pathway is stabilized within a range of genetic and environmental variations. Thus, competitive optimization can also occur in development: as discussed above for RNA, selection for a new target increases the fitness of a previously suboptimal phenotype. Genetic diversification increases the prevalence of this phenotype, a canalization that the system can produce without the new selection pressure. Subsequently, further diversification may occur.

The evolution of modified organs under perturbation suggests that organs may have evolved initially through cooption of core processes into new modules. Larval organs may be coopted piecemeal from a direct developmental pathway, initially as facultative variations but later as a constitutive pathway with metamorphosis after the larval stage (Sly et al., 2003; Raff, 2008). Or, structures may evolve that use coopted core processes later in development than their initial use. For example, in vertebrate embryos the patterning of appendages uses the Hox complex of genes, which is earlier expressed along the anterior-posterior axis of the body and in the pharyngeal arches (Tabin et al., 1999; Minelli, 2000). The capacity to generate, pattern, and differentiate a novel organ may evolve through competitive optimization.

Some transgenerational epigenetic changes may have evolved through competitive optimization, in ways analogous to changes in development. A new selection pressure, within an organism or from outside it, might encourage alternative epigenetic ways to deal with that pressure. These could diversify, be refined, and combine to give a new module for dealing with the pressure. Structural templating in prions is epigenetic, though not heritable. A prion may evolve when a change in selection pressure favors a physiological response that previously was atypical. Mutations of a protein that promote this response may stabilize an alternative configuration of the protein (Schmitt-Ulms et al., 2009; Ehsani et al., 2011; Gendoo and Harrison, 2011). A heritable epigenetic change could evolve when mutations promote enzymatic modification (e.g., by methylation of bases or acetylation of histones) of genes that contribute to a previously atypical response. Small noncoding RNA (sncRNA) can also contribute to heritable epigenetic regulation, and it may evolve through competitive optimization. Of interest here are sncRNAs that bind to a partner—to DNA, other RNAs, or proteins. The sequence of a sncRNA and the regulation of its transcription may vary. If the variation is deleterious to fitness, selection is likely to block its effect. If the variation is beneficial, further variants can promote transcription in situations where it is favorable, or stabilize binding by sequence changes in the sncRNA or its partner. Other molecules may evolve to act in synergy with the sncRNA. The net effect of these changes would be the formation of a new module encompassing transcription, synergistic cooperation, and binding to partners in favorable situations. This process may have contributed to the evolution of diverse sncRNAs—tRNAs, snoRNAs, microRNAs, siRNAs, and piRNAs.

## Discussion

We have proposed a linkage hypothesis to explain the existence of biphasic patterns of diversification in evolution. If a part starts to perform a function that increases the fitness of a system (e.g., an organism), variants of the part diversify. Competition among variants with optimization of functioning restricts the set of surviving parts. These parts are linked in modules and regulatory circuits that promote robust functioning. The modules are available for reuse in new variants and combinations, allowing a second phase of diversification.

Competitive optimization includes both variation and selection and is broader in concept than natural selection. A change in the environment may stimulate variation through mutation and reassortment of genes; unstimulated variation also occurs. Variants may self-organize, as in folding of proteins and nucleic acids, associations among macromolecules, and morphogenesis of embryos (Newman and Comper, 1990). Differential stability of variants in competition selects among them. Variation and selection can build a hierarchy of modules, often through biphasic diversification. In this process, links may be lost as well as gained. For example, in the evolution of proteins there is a tradeoff between stability and function; links that promote stability may be lost as links that promote function are gained (Caetano-Anollés and Mittenthal, 2010). The resulting modules cooperate, converting free energy to work that is used to build and maintain the system (very much as an engine; Cottrell, 1979).

Competitive optimization may mediate the evolution of innovations—the coalescence of frozen accidents characteristic of biological organization. We have offered examples at the molecular, cellular, and developmental levels; many other major transitions occurred at these levels (Szathmáry and Smith, 1995; Kirschner and Gerhart, 2005; Jablonka and Lamb, 2006). Competitive optimization may also occur in macroevolution, as a new species or higher-level taxon arises.

### Linkage hypotheses for developmental and evolutionary biphasic patterns

A biphasic pattern of diversity—an hourglass—often occurs in development: the embryos of a taxon are more similar at a phylotypic stage than earlier or later (Slack et al., 1993). Before the phylotypic stage, early development occurs in various contexts of support and protection—in eggs with various amounts of yolk and lipids, and in various kinds of placentas. The positional information for the axes of the embryo is set up in diverse ways. Diverse paths of early development can converge to the same phylotypic stage through shared core processes (Jessell and Melton, 1992; Kirschner and Gerhart, 2005). Later, organ primordia differentiate into organs. As evolution proceeds, a primordium may follow diverse paths of development; vertebrate appendage buds may generate fins, flippers, legs, arms, and the wings of birds and bats. Thus, the developmental trajectories of related embryos can be represented as a bundle in the shape of an hourglass: after fertilization the trajectories are diverse, but they converge toward a phylotypic stage; subsequently they diverge. A phylotypic stage should be regarded as a period rather than a narrowly defined stage, and the similarities among embryos are qualitative rather than quantitative (Richardson et al., 1997).

Sander (1983) and Raff (1996) interpreted developmental hourglasses in terms of linkage. Early in development, linkage may be strong within spatially distributed molecular networks that produce the embryonic axes and in the networks that support development, but weak between networks. As an embryo approaches the phylotypic stage its cells interact extensively. Linkage increases as cell groups signal to each other in the process of embryonic induction, and inhibitory interactions limit the extent of inductions. Subsequently, during organogenesis, linkage is strong within organs, although reuse of molecules for signaling or cell–cell interaction in various contexts (pleiotropy) links organs indirectly. Linkage between organs tends to be loose, allowing multiple paths of organ development. Processes within one organ have little effect on another until integration among organs' activities occurs, as regulatory systems (neural, endocrine, and immune) unify the organs into a functioning organism.

Such a developmental hourglass operates once in the lifetime of each organism. A developmental hourglass can also occur at the cellular level, repeated in each cell cycle. In prophase, pairs or tetrads of condensed chromosomes are diversely distributed within a cell. Microtubules gather the chromosomes onto a metaphase plate, a relatively invariant structure. Subsequent events are diverse—homologous chromosomes or sister chromatids may separate; a cell may cleave to two daughters or remain uncleaved.

Cells bring each chromosome to an equivalent position through exploratory behavior of microtubules, as Kirschner and Gerhart (2005) explain. A biphasic process can occur repeatedly within a life cycle, as an organism uses exploration to solve a problem and then may use the solution in various ways. The organism may repeat a behavior each time it solves a given problem, as ants do in seeking food. Or, it may learn to solve the problem in a single trial through physical or mental exploration. With knowledge of causal relations, an organism can envision alternative pathways to an outcome (Gopnik, 2009). A causal relation is analogous to a biochemical reaction: making allowed connections in a repertoire of reactions allows the evolution of alternative biochemical pathways, as discussed above for the tricarboxylic acid cycle. Thus biphasic patterns occur on several time scales, with various degrees of repetition.

Note that a developmental hourglass need not evolve through competitive optimization. Kirschner and Gerhart (2005) suggested that processes generating a phylotypic stage, including axis specification and compartmentation, could evolve earlier than the diversifications before and after that stage. Newman (2011) has further elaborated on this concept for the origin of the egg stage of animal development, with eggs representing sets of independent evolutionary innovations inserted into the developmental trajectories of ancient aggregates of cells ultimately responsible for different body plans. However, in competitive optimization the first phase of diversification must precede and allow the consolidation into the canalized stage. Thus, a developmental hourglass does not necessarily arise through an evolutionary biphasic pattern.

The linkage hypothesis for developmental hourglasses initiated our linkage hypothesis for evolutionary hourglasses, so it is important to clarify relations between these hourglasses. In an evolutionary hourglass, a biphasic change in the rate of diversification occurs only once in the entire course of evolution. However, a developmental hourglass recurs once in each developing embryo or cell cycle. A behavioral hourglass may occur once or repeatedly in the life cycle of an organism. A developmental hourglass bundles the diverse developmental trajectories of a group of related embryos. By contrast, an evolutionary hourglass simply tallies the number of parts existing at a sequence of times, without presenting trajectories between the parts. In both kinds of hourglasses, exploration among alternatives may occur during diversification. In both kinds, the spatial distribution of diversification may be wide during the early phase, but regionally localized in the late phase; an example of the latter in an evolutionary hourglass is the formation of localized modules within evolving RNA molecules (Ancel and Fontana, 2000).

### Other interpretations of biphasic diversification

In the competitive optimization hypothesis, variants of a structure compete for a finite set of functional niches. Competition limits the rate at which new variants survive, ending the first phase in a biphasic pattern. This rate might also be limited because the set of possible variants is limited, and the process of diversification exhausts the set. After the first phase, new processes of mutation might evolve to enlarge the set and allow further diversification. Exhaustion may have contributed to the biphasic pattern of FSF diversification, along with competitive optimization. The rate at which biologists have found new FSFs suggests that there are only a few thousand of them (less than 3400 FSFs; Levitt, 2007). At a given time, available processes of mutation may limit the transitions between FSFs, limiting the appearance of new FSFs during the first phase. It is not evident whether exhaustion of variants would contribute to an increase in linkage.

A biphasic pattern of evolutionary diversification might result from causes endogenous or exogenous to organisms. A decline in the rate of FSF generation may have resulted from processes endogenous to cells—competition among FSFs for functional niches and selection pressure for cooperation—but also from competition among cells for resources. A bottleneck—a major restriction in diversity—can occur without formation of a module if the environment of a diversifying population undergoes a major change. The survivors will initially display less diversity than their predecessors, though they are likely to diversify subsequently. Well-known examples include the diversification of dinosaurs after the post-Permian extinction 0.25 Gyr ago and of mammals after the post-Cretaceous extinction 0.065 Gyr ago.

An exogenous factor, the increase in atmospheric oxygen resulting from photosynthesis, may have reduced the rate of FSF diversification after its first peak, 2.6 Gyr ago. This decline occurred as the atmospheric oxygen level was increasing above 0.1% of the present atmospheric level (PAL). The increase to 1% PAL was probably gradual over roughly 400 million years, from about 2.9–2.45 Ga ago (Wang et al., 2011). During this interval oxygen would have been toxic to many species, decreasing their production of new FSFs while giving new opportunities for new FSF alternatives.

A sufficiently sudden and severe exogenous stress extinguishes many species, opening niches for new diversification. A more gradual stress imposes selection pressures that can increase linkage in the survivors, allowing a new integration of evolving diversity. The increase in oxygen level provided challenges and opportunities through which more complex cells evolved. Many metal-binding FSFs evolved (Dupont et al., 2010) and were used in carriers, enzymes and transcription factors that aided the response to oxygen. FSFs associated in new metabolic pathways. Pathways using or producing oxygen became localized within compartments—chloroplasts, mitochondria, and peroxisomes. New gene regulatory networks expressed proteins in new functional contexts. The magnitude of this response is evident in the facultative anaerobe *Saccharomyces cerevisiae* (Lai et al., 2006). This yeast has 6607 open reading frames (*Saccharomyces* Genome Database, February 2011). In galactose, after transitions from aerobic to anaerobic conditions and back, the expression levels of 2388 genes change. Reoxygenation affects genes dealing with oxidative stress, redox regulation, respiration, perioxisome function, lipid metabolism, sulfur metabolism, metal ion homeostasis and biosynthesis of unsaturated fatty acids, heme, thiamine, homocysteine, and S-adenosyl methionine. Thus, both exogenous and endogenous factors can contribute to the increase in linkage posited in the competitive optimization hypothesis. At present it is unclear how to dissect their relative contributions.

### Limitations, alternatives, and tests for the linkage hypothesis

Several hypotheses may explain an evolutionary hourglass. In our linkage hypothesis, competition among diversifying parts increases linkage among surviving parts, which form a module. Later the module may diversify and be used in diverse contexts. There are alternative hypotheses: Modules can evolve without selection as well as under indirect or direct selection (Wagner et al., 2007). Diversification preceding the formation of a module may be unrelated to its formation, as discussed for developmental hourglasses. A bottleneck in diversity can occur without formation of a module if the environment changes greatly.

It is desirable to distinguish among these alternatives. Testing might occur through *in vitro* evolution of macromolecules or *in vivo* evolution of cells, sometimes in synthetic biology settings. It is also desirable to predict the circumstances in which alternative processes are likely to generate an hourglass. Increasing linkage may accompany diversification in some situations, but not others; what determines the correlation? To address this issue, knowledge of mechanisms producing diversification and linkage is necessary. Various mechanisms can produce an hourglass. Dynamical models for mechanisms, explored with analysis and computer simulation, could relate mechanisms to outcomes. Models could show more explicitly how competitive optimization occurs, and systematize and rationalize its occurrence.

To test a linkage hypothesis it is necessary to formalize the concept of linkage. In a network where nodes represent parts, the extent and pattern of connectivity among nodes provides indices of linkage. One can also quantify the information in the system beyond the information in its parts, and so measure how much the state of each part affects the state of other parts (Tononi, 2008).

## Conclusion

Understanding patterns of evolutionary change is challenging. In this paper we suggest that biphasic patterns of diversification can evolve through competitive optimization. In this process, diversifying variants of a system converge under selection to a cohesive unit, a module, which subsequently diversifies. Thus, unification occurs through diversification and provides the basis for subsequent diversification. We believe this process occurred widely, in the evolution of macromolecules, networks, cells, and multicellular development, and may still be generating hierarchical complexity in life. Future modeling and data mining endeavors can test this hypothesis and assess its place in the physics of systems far from equilibrium.

### Conflict of interest statement

The authors declare that the research was conducted in the absence of any commercial or financial relationships that could be construed as a potential conflict of interest.
